# Research on the Necessity of Lie Group Strapdown Inertial Integrated Navigation Error Model Based on Euler Angle

**DOI:** 10.3390/s22207742

**Published:** 2022-10-12

**Authors:** Leiyuan Qian, Fangjun Qin, Kailong Li, Tiangao Zhu

**Affiliations:** College of Electrical Engineering, Naval University of Engineering, Wuhan 430033, China

**Keywords:** integrated navigation, lie group, necessity of research, variance estimation, observability

## Abstract

In response to the lack of specific demonstration and analysis of the research on the necessity of the Lie group strapdown inertial integrated navigation error model based on the Euler angle, two common integrated navigation systems, strapdown inertial navigation system/global navigation satellite system (SINS/GNSS) and strapdown inertial navigation system/doppler velocity log (SINS/DVL), are used as subjects, and the piecewise constant system (PWCS) matrix, based on the Lie group error model, is established. From three aspects of variance estimation, the observability and performance of the system with large misalignment angles for low, medium, and high accuracy levels, traditional error model, Lie group left error model, and right error model are compared. The necessity of research on Lie group error model is analyzed quantitatively and qualitatively. The experimental results show that Lie group error model has better stability of variance estimation, estimation accuracy, and observability than traditional error model, as well as higher practical value.

## 1. Introduction

Integrated navigation systems have become one of the research hotspots in the field of navigation, which are mainly SINS and supplemented by GNSS, DVL, odometer (OD), and other systems [1,2,3,4]. A reasonable model is one of the keys for the integrated navigation system to accomplish navigation task. The integrated navigation models mainly include two categories: direct navigation model and indirect navigation model [5]. The direct navigation model is nonlinear, which uses the navigation parameters (attitude, velocity, position, etc.) as the state, but has a high operational complexity [6,7]. The indirect navigation model is mostly linear, which uses navigation parameter errors (attitude error, velocity error, position error, etc.) as state quantities and has been used in a variety of applications [8,9,10].

Indirect integrated navigation error models mainly include strapdown inertial integrated navigation error model based on Euler angle and quaternion [11,12]. The Euler angle errors is used as an attitude error state of strapdown inertial integrated navigation error model, based on Euler angles. Then, after converting to quaternions, the navigation calculation update is performed. Because the filtering equation of the model is linear, the model is more commonly used [13]. The quaternion error is used as an attitude error state of integrated navigation error model, based on quaternion, which can directly participate in navigation calculation updates. However, the application of this model is limited because the filtering equation is nonlinear and has a high dimension [7].

In the traditional integrated navigation error model, attitude, velocity, and position errors are not in same space [14]. As a combination of algebra and geometry, the Lie group extends the solvability of algebraic equations to differential equations [15] and introduces state quantities, such as attitude and velocity, into a group, which constitutes the special Euclidean group (SE(3)) and provides a new idea for the construction of integrated navigation models [16,17]. Ref. [18] applied Lie group theory to the solution of the directional cosine matrix and quadratic differential equations for an inertial navigation system (INS). Ref. [19] achieved the initial alignment of the SINS with a large misalignment angle based on Lie group theory. Ref. [20] derived the quaternion model based on Lie group theory and proved the effect of the model. According to the different of error definitions, the left and right error models of integrated navigation based on Lie group in different coordinate systems were successively proposed [21,22]. Refs. [23,24] introduced Lie group state space into Kalman filter (KF) to make the state space independent of the carrier trajectory and proposed the invariant extended Kalman filter (IEKF). Ref. [25] derived the Lie group model of SINS/GNSS in the nonlinear UKF framework and pointed out that Lie group left error model has certain advantages.

In the field of navigation, researchers have achieved many results in researching Lie group theory. However, regarding the research necessity of the Lie group error model of the integrated navigation based on Euler angle, the advantages and disadvantages, compared with traditional error models, have not been clarified. In this paper, based on the error model of integrated navigation, the PWCS matrix based on Lie group error model is established. The commonly used SINS/GNSS and SINS/DVL integrated navigation systems are used as experimental subjects to quantitatively and qualitatively analyze Lie group error model of integrated navigation from these aspects of variance estimation, large misalignment angle navigation performance, and observability. The necessity for the research of the Lie group model is pointed out and justified, which provides a basis for its application in integrated navigation.

The remainder of this paper is organized as follows. In Section 2, the Lie group error model of integrated navigation based on Euler angle is introduced. In Section 3, the analytical methods of variance estimation and observability are introduced, and the PWCS matrix based on the Lie group model is established. In Section 4, the necessity of Lie group model in variance estimation of integrated navigation is analyzed through simulation experiment. In Section 5, for the SINS/GNSS and SINS/DVL of different accuracy levels, the necessity of the Lie group model is analyzed at large misalignment angles. In Section 6, the observability of the Lie group model in SINS/GNSS and SINS/DVL is analyzed through field test experiments. The corresponding conclusion are given in last section.

## 2. Lie Group Error Model of Integrated Navigation in Navigation Coordinate System

### 2.1. Related Foundations

The common coordinate systems used in integrated navigation are defined as follows. The geocentric inertial coordinate system is called the i-system. The Earth coordinate system is called the e-system. In this paper, the East-North-Up (ENU) geographic coordinate system is used as the ideal navigation coordinate system, called the n-system. The ideal Right-Front-Up (RFU) carrier coordinate system is called the b-system. The term “ideal” means that it does not contain calculation errors.

In practical application, SINS and its integrated navigation systems often are operated under n. Therefore, this paper discusses for the integrated navigation system under n.

The state error of the integrated navigation system is defined in n as follows.

(1) $$x={\left[\begin{array}{lllll} \delta \varphi & \delta v^{n} & \delta p^{n} & \varepsilon^{b} & \nabla^{b} \end{array}\right]}^{T}$$

where δφ is the error of attitude angle, $\delta \varphi =\left[\begin{array}{ccc} \delta \theta & \delta \gamma & \delta \psi \end{array}\right]$. θ, γ, and ψ denote the pitch, roll, and yaw angles of the carrier, respectively. $\delta v^{n}$ is the error of speed, $\delta v^{n}=\left[\begin{array}{ccc} \delta v_{E}^{n} & \delta v_{N}^{n} & \delta v_{U}^{n} \end{array}\right]$. $v_{E}^{n}$, $v_{N}^{n}$, and $v_{U}^{n}$ denote the eastward speed, northward speed, and skyward speed of the carrier, respectively. $\delta p^{n}$ is the error of position, $\delta p^{n}=\left[\begin{array}{ccc} \delta L & \delta \lambda & \delta h \end{array}\right]$. L, λ, and h denote the latitude, longitude, and altitude of the carrier, respectively. $\varepsilon^{b}$ is the drift error of the gyroscope. $\nabla^{b}$ is the drift error of the accelerometer.

According to Lie group theory [26,27,28], SINS states [29] in the special orthogonal group (SO(3)) and Euclidean space can be incorporated into SE(3) to form Lie group states.

(2) $$\chi =\left[\begin{array}{ccc} C_{b}^{n} & v^{n} & p^{n}\\ 0_{1\times 3} & 1 & 0\\ 0_{1\times 3} & 0 & 1 \end{array}\right]$$

where $C_{b}^{n}$ is the directional cosine matrix from b to n.

Then, the models under two different definitions of error are obtained.

(3) $$\eta_{l}={\tilde{\chi }}^{-1}\chi$$

(4) $$\eta_{r}=\chi {\tilde{\chi }}^{-1}$$

where $\eta_{l}$ is the left invariant error, and $\eta_{r}$ is the right invariant error. The “~” indicates parameters with calculation errors calculated by the navigation system or obtained by sensors.

According to the relationship between Lie group and Lie algebra [30], the left and right error state equations of Lie group can be obtained, respectively [31].

### 2.2. Left Error Model

The left error state equation of Lie group is as follows.

(5) $$\dot{x}=F_{l}x+G_{l}W$$

where $F_{l}$ is the state transfer matrix of left error model. $G_{l}$ is the noise transfer matrix of the model. W is the vector of process noise, which is the same as traditional model. The formula for $F_{l}$ is specified as follows.

(6) $$F_{l}=\left[\begin{array}{ccccc} -\left({\tilde{\omega }}_{ib}^{b}\times \right) & -{\tilde{C}}_{n}^{b}M_{2}{\tilde{C}}_{b}^{n} & {\tilde{C}}_{n}^{b}\left(M_{1}+M_{2}\right){\tilde{C}}_{b}^{n} & -I_{3\times 3} & 0_{3\times 3}\\ -\left({\tilde{f}}_{ib}^{b}\times \right) & {\tilde{C}}_{n}^{b}\left({\tilde{v}}^{n}\times \right)M_{2}{\tilde{C}}_{b}^{n}-\left({\tilde{\omega }}_{ib}^{b}\times \right)-{\tilde{C}}_{n}^{b}\left({\tilde{\omega }}_{ie}^{n}\times \right){\tilde{C}}_{b}^{n} & {\tilde{C}}_{n}^{b}\left({\tilde{v}}^{n}\times \right)\left(2M_{1}+M_{3}\right) & 0_{3\times 3} & -I_{3\times 3}\\ 0_{3\times 3} & -N_{rv}{\tilde{C}}_{b}^{n} & N_{rr} & 0_{3\times 3} & 0_{3\times 3}\\ 0_{3\times 3} & 0_{3\times 3} & 0_{3\times 3} & 0_{3\times 3} & 0_{3\times 3}\\ 0_{3\times 3} & 0_{3\times 3} & 0_{3\times 3} & 0_{3\times 3} & 0_{3\times 3} \end{array}\right]$$

where $M_{1}$, $M_{2}$, $M_{3}$, $N_{rv}$, and $N_{rr}$ are, respectively,

(7) $$M_{1}=\left[\begin{array}{ccc} 0 & 0 & 0\\ 0 & \frac{-\omega_{ie}\sin L}{R_{M}+h} & 0\\ 0 & \frac{\omega_{ie}\cos L}{R_{M}+h} & 0 \end{array}\right],\;M_{2}=\left[\begin{array}{ccc} 0 & -\frac{1}{R_{M}+h} & 0\\ \frac{1}{R_{N}+h} & 0 & 0\\ \frac{\tan L}{R_{N}+h} & 0 & 0 \end{array}\right],\;M_{3}=\left[\begin{array}{ccc} 0 & 0 & \frac{v_{N}^{n}}{{\left(R_{M}+h\right)}^{2}}\\ 0 & 0 & -\frac{v_{E}^{n}}{{\left(R_{N}+h\right)}^{2}}\\ 0 & \frac{v_{E}^{n}}{\left(R_{N}+h\right)\left(R_{M}+h\right)\cos^{2}L} & -\frac{v_{E}^{n}\tan L}{{\left(R_{N}+h\right)}^{2}} \end{array}\right]$$

(8) $$N_{rv}=\left[\begin{array}{ccc} 0 & \frac{1}{R_{M}+h} & 0\\ \frac{1}{\left(R_{N}+h\right)\cos L} & 0 & 0\\ 0 & 0 & 1 \end{array}\right],\;N_{rr}=\left[\begin{array}{ccc} 0 & 0 & -\frac{v_{N}^{n}}{{\left(R_{M}+h\right)}^{2}}\\ \frac{v_{E}^{n}\tan L}{\left(R_{N}+h\right)\cos L} & 0 & -\frac{v_{E}^{n}}{{\left(R_{N}+h\right)}^{2}\cos L}\\ 0 & 0 & 0 \end{array}\right]$$

The formula for $G_{l}$ is specified as follows.

(9) $$G_{l}=\left[\begin{array}{cc} -I_{3\times 3} & 0_{3\times 3}\\ 0_{3\times 3} & -I_{3\times 3}\\ 0_{3\times 3} & 0_{3\times 3} \end{array}\right]$$

where $\omega_{ib}^{b}$ is the increment of angular speed output from the gyroscope. $R_{N}$ is the radius of curvature in prime vertical. $R_{M}$ is the radius of curvature in meridian. $\omega_{ie}$ is the angular speed of the earth rotation. $f_{ib}^{b}$ is the specific force. (·×) denotes the skew-symmetric, taking $\left({\tilde{v}}^{n}\times \right)$ as an example. ${\tilde{v}}^{n}={\left[\begin{array}{ccc} {\tilde{v}}_{E}^{n} & {\tilde{v}}_{N}^{n} & {\tilde{v}}_{U}^{n} \end{array}\right]}^{T}$, then $\left({\tilde{v}}^{n}\times \right)=\left[\begin{array}{ccc} 0 & -{\tilde{v}}_{U}^{n} & {\tilde{v}}_{N}^{n}\\ {\tilde{v}}_{U}^{n} & 0 & -{\tilde{v}}_{E}^{n}\\ -{\tilde{v}}_{N}^{n} & {\tilde{v}}_{E}^{n} & 0 \end{array}\right]$.

Correspondingly, in the left error model, the navigation parameters can be corrected by the following equation.

(10) $$\{\begin{array}{l} C_{b,,update}^{n,}={\tilde{C}}_{b}^{n}\exp\left(\delta \varphi \right)\\ v_{update}^{n}={\tilde{v}}^{n}+{\tilde{C}}_{b}^{n}\delta v^{n}\\ p_{update}^{n}={\tilde{p}}^{n}-\delta p^{n} \end{array}$$

### 2.3. Right Error Model

The right error state equation of Lie group is as follows.

(11) $$\dot{x}=F_{r}x+G_{r}W$$

where $F_{r}$ and $G_{r}$ are the state transfer matrix and noise transfer matrix of right error model, respectively. W is the same as traditional model and left error model.

The formula for $F_{r}$ is specified as follows.

(12) $$F_{r}=\left[\begin{array}{ccccc} -N_{2}\left({\tilde{v}}^{n}\times \right)-{\tilde{\omega }}_{in}^{n} & -N_{2} & N_{1}+N_{3} & -{\tilde{C}}_{b}^{n} & 0_{3\times 3}\\ \left({\tilde{v}}^{n}\times \right)\left({\tilde{\omega }}_{ie}^{n}\times \right)+\left({\tilde{g}}^{n}\times \right) & \left(2{\tilde{\omega }}_{ie}^{n}+{\tilde{\omega }}_{en}^{n}\right)\times & -\left({\tilde{v}}^{n}\times \right)N_{1} & \left({\tilde{v}}^{n}\times \right){\tilde{C}}_{b}^{n} & -{\tilde{C}}_{b}^{n}\\ N_{rv}\left({\tilde{v}}^{n}\times \right) & -N_{rv} & N_{rr} & 0_{3\times 3} & 0_{3\times 3}\\ 0_{3\times 3} & 0_{3\times 3} & 0_{3\times 3} & 0_{3\times 3} & 0_{3\times 3}\\ 0_{3\times 3} & 0_{3\times 3} & 0_{3\times 3} & 0_{3\times 3} & 0_{3\times 3} \end{array}\right]$$

where $N_{1}$ and $N_{2}$ are, respectively,

(13) $$N_{1}=\left[\begin{array}{ccc} 0 & 0 & 0\\ -\omega_{ie}\sin L & 0 & 0\\ \omega_{ie}\cos L & 0 & 0 \end{array}\right],\;N_{2}=M_{2},\;N_{3}=\left[\begin{array}{ccc} 0 & 0 & \frac{v_{N}^{n}}{{\left(R_{M}+h\right)}^{2}}\\ 0 & 0 & -\frac{v_{E}^{n}}{{\left(R_{N}+h\right)}^{2}}\\ \frac{v_{E}^{n}}{\left(R_{N}+h\right)\cos^{2}L} & 0 & -\frac{v_{E}^{n}\tan L}{{\left(R_{N}+h\right)}^{2}} \end{array}\right]$$

The formula for $G_{r}$ is specified as follows.

(14) $$G_{r}=\left[\begin{array}{cc} -{\tilde{C}}_{b}^{n} & 0_{3\times 3}\\ -\left({\tilde{v}}^{n}\times \right){\tilde{C}}_{b}^{n} & -{\tilde{C}}_{b}^{n}\\ 0_{3\times 3} & 0_{3\times 3} \end{array}\right]$$

where $\omega_{in}^{n}$ is the angular speed of the motion in n-coordinate system, $\omega_{in}^{n}=\omega_{ie}^{n}+\omega_{en}^{n}$. $\omega_{en}^{n}$ is the implicated angular speed caused by the motion of carrier.

Correspondingly, in the right error model, the correction formula for the navigation parameters is as follows.

(15) $$\{\begin{array}{l} C_{b,,update}^{n,}=\exp\left(\delta \varphi \right){\tilde{C}}_{b}^{n}\\ v_{update}^{n}={\tilde{v}}^{n}-\left({\tilde{v}}^{n}\times \right)\delta \varphi +\delta v^{n}\\ p_{update}^{n}={\tilde{p}}^{n}-\delta p^{n} \end{array}$$

### 2.4. Observation Equations for Commonly Used Integrated Navigation Systems

For the left and right error models, the observation equations are divided into the left invariant and right invariant.

The speed and position are usually used as observations of SINS/GNSS. The left invariant observation equation of integrated system is as follows.

(16) $$z_{SINS/GNSS}=\left[\begin{array}{c} \Delta v_{SINS/GNSS}\\ \Delta p_{SINS/GNSS} \end{array}\right]=H_{l}^{SINS/GNSS}x+V_{SINS/GNSS}$$

(17) $$H_{l}^{SINS/GNSS}=\left[\begin{array}{lllll} 0_{3\times 3} & -{\tilde{C}}_{b}^{n} & 0_{3\times 3} & 0_{3\times 3} & 0_{3\times 3}\\ 0_{3\times 3} & 0_{3\times 3} & I_{3\times 3} & 0_{3\times 3} & 0_{3\times 3} \end{array}\right]$$

where $\Delta v_{SINS/GNSS}$ is the difference of the speed provided by SINS and GNSS. $\Delta p_{SINS/GNSS}$ is the difference of the position. $V_{SINS/GNSS}$ is the matrix of observation noise, which is the same as the traditional SINS/GNSS model.

The right invariant observation equation is as follows.

(18) $$z_{SINS/GNSS}=\left[\begin{array}{c} \Delta v\\ \Delta p \end{array}\right]=H_{r}^{SINS/GNSS}x+V_{SINS/GNSS}$$

(19) $$H_{r}^{SINS/GNSS}=\left[\begin{array}{lllll} \left({\tilde{v}}^{n}\times \right) & -I_{3\times 3} & 0_{3\times 3} & 0_{3\times 3} & 0_{3\times 3}\\ 0_{3\times 3} & 0_{3\times 3} & I_{3\times 3} & 0_{3\times 3} & 0_{3\times 3} \end{array}\right]$$

The difference of speed is often used as an observation of SINS/DVL. The left invariant observation equation of SINS/DVL is as follows.

(20) $$z_{SINS/DVL}=\left[\Delta v_{SINS/DVL}\right]=H_{l}^{SINS/DVL}x+V_{SINS/DVL}$$

(21) $$H_{l}^{SINS/DVL}=\left[\begin{array}{lllll} -\left({\tilde{v}}^{n}\times \right) & -{\tilde{C}}_{b}^{n} & 0_{3\times 3} & 0_{3\times 3} & 0_{3\times 3} \end{array}\right]$$

where $\Delta v_{SINS/DVL}$ is the difference between the speed information provided by SINS and DVL. $V_{SINS/DVL}$ is the matrix of observation noise, which is the same as traditional SINS/DVL model.

The right invariant observation equation is as follows.

(22) $$z_{SINS/DVL}=\left[\Delta v_{SINS/DVL}\right]=H_{r}^{SINS/DVL}x+V_{SINS/DVL}$$

(23) $$H_{r}^{SINS/DVL}=\left[\begin{array}{lllll} 0_{3\times 3} & -I_{3\times 3} & 0_{3\times 3} & 0_{3\times 3} & 0_{3\times 3} \end{array}\right]$$

## 3. Method of Analysis of Necessity

In this paper, the necessity of the Lie group model, in terms of variance estimation stability, observability, and estimation performance of SINS with different accuracy in misalignment angle environment, is analyzed. The relevant analytical methods are described as follows.

### 3.1. Analytical Method of Variance

KF is commonly used to estimate the state of the integrated navigation system [32]. The mean square error matrix $P_{k}$ of KF reflects the strength of the estimated state, which is one of the criteria for judging the performance of the integrated navigation filter. The equation of $P_{k}$ is as follows.

(24) $$P_{k}=E\left[{\tilde{X}}_{k}{\tilde{X}}_{k}^{T}\right]=\left[\begin{array}{cccc} E\left[{\left({\tilde{X}}_{k}^{\left(1\right)}\right)}^{2}\right] & E\left[\left({\tilde{X}}_{k}^{\left(1\right)}{\tilde{X}}_{k}^{\left(2\right)}\right)\right] & \cdots & E\left[\left({\tilde{X}}_{k}^{\left(1\right)}{\tilde{X}}_{k}^{\left(n\right)}\right)\right]\\ E\left[\left({\tilde{X}}_{k}^{\left(2\right)}{\tilde{X}}_{k}^{\left(1\right)}\right)\right] & E\left[{\left({\tilde{X}}_{k}^{\left(2\right)}\right)}^{2}\right] & \cdots & E\left[\left({\tilde{X}}_{k}^{\left(2\right)}{\tilde{X}}_{k}^{\left(n\right)}\right)\right]\\ \vdots & \vdots & \vdots & \vdots \\ E\left[\left({\tilde{X}}_{k}^{\left(n\right)}{\tilde{X}}_{k}^{\left(1\right)}\right)\right] & E\left[\left({\tilde{X}}_{k}^{\left(n\right)}{\tilde{X}}_{k}^{\left(2\right)}\right)\right] & \vdots & E\left[{\left({\tilde{X}}_{k}^{\left(n\right)}\right)}^{2}\right] \end{array}\right]$$

where ${\tilde{X}}_{k}=X_{k}-{\hat{X}}_{k}$. ${\hat{X}}_{k}$ is the state estimate of the system at moment k. $X_{k}$ is the true state of the system at moment k. ${\tilde{X}}_{k}^{\left(l\right)}$ (l=1,2,⋯,n) is the lth component of ${\tilde{X}}_{k}$. n is the number of state dimensions. $E\left[{\left({\tilde{X}}_{k}^{\left(1\right)}\right)}^{2}\right]$, $E\left[{\left({\tilde{X}}_{k}^{\left(2\right)}\right)}^{2}\right]$, ⋯, $E\left[{\left({\tilde{X}}_{k}^{\left(n\right)}\right)}^{2}\right]$ are the variance of each state. The non-diagonal elements of $P_{k}$ are the covariances between states.

The covariance is used to measure the relationship between states. The variance is used to reflect the degree of dispersion of the corresponding state.

The minimum of the estimation error is the minimum of the sum of the mean square error. The estimation effect of integrated navigation can be judged by the following formula.

(25) $$E\left[{\left({\tilde{X}}_{k}^{\left(1\right)}\right)}^{2}\right]+E\left[{\left({\tilde{X}}_{k}^{\left(2\right)}\right)}^{2}\right]+\cdots +E\left[{\left({\tilde{X}}_{k}^{\left(n\right)}\right)}^{2}\right]=\min$$

The larger the variance, the bigger the difference between the estimated state and true value. The smaller the variance, the smaller the difference between the estimated state and true value, and the better the estimation [33].

In integrated navigation, the variance of the traditional model is inconsistent, that is, the variance varies for different states of motion. However, this is not reasonable. Under ideal conditions, such as static, it is difficult for the variance of integrated navigation to achieve the perfect state [34]. In particular, those states that are not related to observations should be given further attention. This is because, for KF, the greater the correlation of the observations, the better the estimation of the state. Additionally, the state associated with the observation can be better estimated. The navigation parameters of SINS can be corrected by the corresponding observation information, constraining its divergence. However, the observation-independent parameters are difficult to effectively estimate. For example, for an integrated navigation system, in which speed and position are the observed information, the effects of the attitude, accelerometer, and gyroscope state estimations should be paid more attention.

Therefore, we can extract the variance of the corresponding states from $P_{k}$ and analyze the performance of different integrated navigation models in different motion states.

### 3.2. Analytical Method of Observability

The observability of states is one of the criteria for judging the advantages or disadvantages of integrated navigation system [35,36]. Both SINS/GNSS and SINS/DVL are time-varying systems, which can be regarded as PWCSs. Thus, we can construct the PWCS observable matrix and use the singular value decomposition (SVD) method to analyze the observability of the integrated navigation system.

The PWCS matrix of integrated navigation system is constructed as follows.

(26) $$\left\{ \begin{array}{l} X_{k}=FX_{k-1}\\ Z_{k}=HX_{k-1} \end{array}\right.$$

where F and H are the state transfer matrix and observation matrix, respectively, which are constant in a very short period of time.

Assuming that the system has the following observation information at this time,

(27) $$\left\{ \begin{array}{l} Z\left(1\right)=HX\left(1\right)\\ Z\left(2\right)=HX\left(1\right)\\ Z\left(3\right)=HX\left(2\right)\\ \vdots \\ Z\left(N\right)=HX\left(N\right) \end{array}\right.$$

According to the recurrence relation of Equation (26), the above equation can be rewritten as

(28) $$\left\{ \begin{array}{l} Z\left(1\right)=HX\left(1\right)\\ Z\left(2\right)=HFX\left(1\right)\\ Z\left(3\right)=HF^{2}X\left(1\right)\\ \vdots \\ Z\left(N\right)=HF^{N-1}X\left(1\right) \end{array}\right.$$

Equation (28) can be further written as

(29) Z=QX(1)

where Q is the discretized PWCS matrix of integrated navigation system at that moment, whose specific expression is

(30) $$Q=\left[\begin{array}{l} H\\ HF\\ HF^{2}\\ \vdots \\ HF^{N-1} \end{array}\right]$$

SVD decomposition of Q is as follows.

(31) $$Q=U\sum V^{T}$$

where U and V are the orthogonal matrix, and $\sum =\left[\begin{array}{c} S\\ 0_{\left(m-r\right)\times r} \end{array}\right]$. S is a diagonal matrix that consists of the singular values $\sigma_{i}$ (i=1,2,⋯,r) of Q. m and r are the dimension of observation and the rank of Q, respectively

Substituting Equation (31) into Equation (29), we can obtain

(32) $$Z=\sum_{i=1}^{r}\sigma_{i}\left(v_{i}^{T}X\left(1\right)\right)u_{i}$$

where $v_{i}$ and $u_{i}$ are the element of V and U, respectively.

When Z contains a constant parametric value, X(1) can form an ellipsoid, and Equation (32) can be expressed as

(33) $${\left|Z\right|}^{2}=\sum_{i=1}^{r}{\left(\frac{v_{i}^{T}X\left(1\right)u_{i}}{\alpha_{i}}\right)}^{2}$$

where $\alpha_{i}=1/\sigma_{i}$ is the length of the principal semi-axis of the ellipsoid.

The number of valid states in integrated navigation is reflected by r. Additionally, the estimated effect of the corresponding state is reflected by the size of $\sigma_{i}$. The larger the $\sigma_{i}$, the better the estimation, and the smaller the $\sigma_{i}$, the worse the estimated effect [37].

For SINS/GNSS, the Equations (6) and (17) at a certain moment can be introduced into Equation (30) to obtain a PWCS matrix based on Lie group left error model. Additionally, Equations (12) and (19) can be introduced into Equation (30) to obtain PWCS matrix based on the Lie group right error model.

For SINS/DVL, Equations (6) and (21), at a certain moment, can be introduced into Equation (30) to obtain PWCS matrix based on Lie group left error model. Equations (12) and (23) can be introduced into Equation (30) to obtain a PWCS matrix based on the Lie group right error model.

## 4. Experiment and Analysis about Variance Estimation

To analyze the necessity of the Lie group model in integrated navigation variance estimation, the variance estimation effects of the traditional error, Lie group left error, and Lie group right error models are quantitatively analyzed under both static and dynamic conditions in the section, using SINS/GNSS as subjects. For comparison, the above three models are denoted as the SO model, LSE model, and RSE model, respectively.

### 4.1. Static Simulation

The static simulation parameters of SINS/GNSS are set as follows.

**Figure 1:**
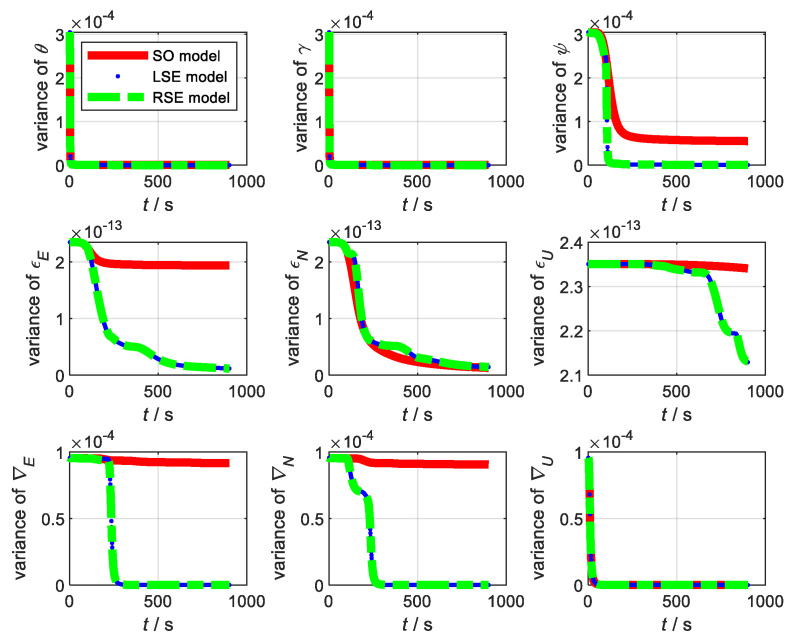
State variance under static condition.

Duration of the simulation, T=900 s. The sampling frequency of GNSS $f_{GNSS}=1\;Hz$. The relevant parameters of the SINS are shown in Table 1. The sampling frequency of SINS $f_{SINS}=10\;Hz$. The sampling frequency of GNSS $f_{GNSS}=1\;Hz$. The drift of the gyroscope was 0.1°/h. The drift of the accelerometer was 0.4 mg. Kalman filter was adopted as the data fusion algorithm for the three models. In SINS/GNSS, the estimated state variance independent of the observations under static conditions are shown in Figure 1. From Figure 1, we can see that the estimations of the variance of the LSE model and RSE model under static conditions were better than SO model. We can conclude that the difference between the estimated states of the Lie group model and true value is small. Therefore, the Li group model under static condition is more applicable to an integrated navigation system than the traditional model.

### 4.2. Dynamic Simulation

**Figure 2:**
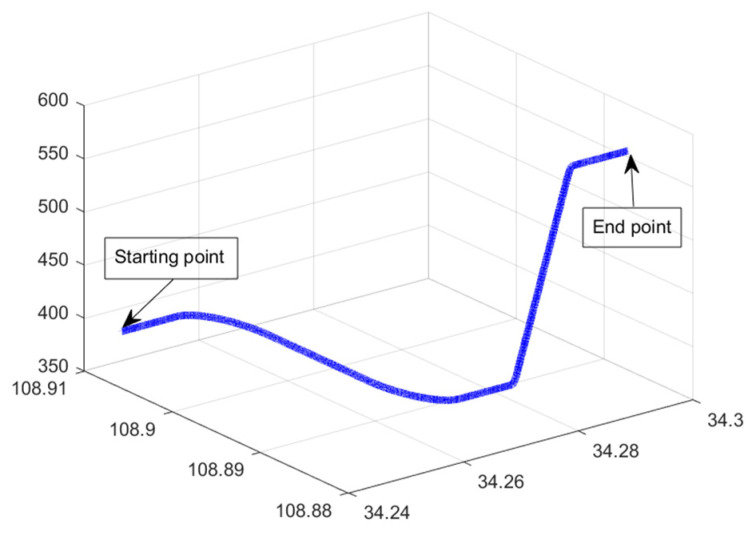
Trajectory of flight.

**Figure 3:**
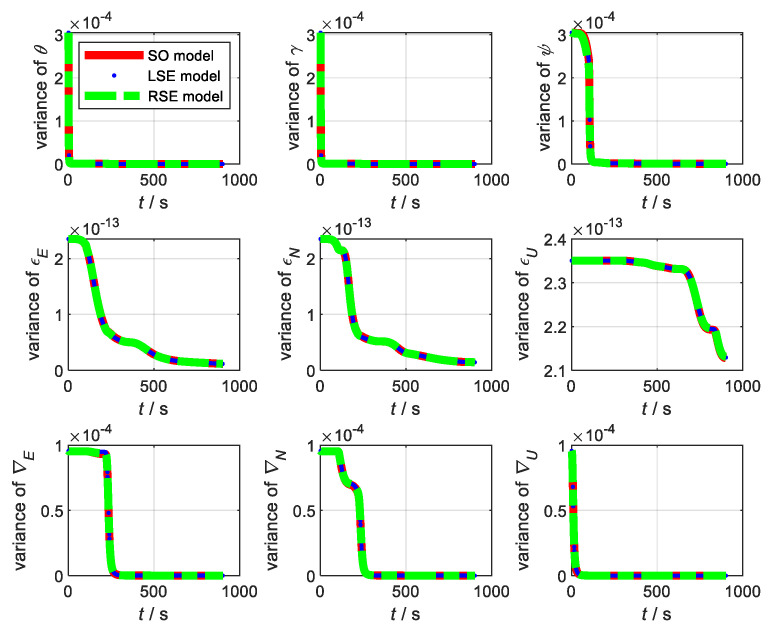
State variance under flight condition.

Next, the flight simulation of SINS/GNSS was performed. The experimental parameters are set in accordance with the static test. The trajectory of flight is shown in Figure 2. The state variance is shown in Figure 3. From Figure 3, the variance estimation effect of SO model under flight condition is almost the same as that of LSE model and RSE model, namely the difference between the estimated states and the true values of the three models is consistent. Therefore, traditional model and Lie group model are applicable to integrated navigation system under flight conditions.

Combining the variance estimation effects of each model under static and flight conditions, we can find that the variance estimated by Lie group error model is almost consistent under static or dynamic conditions, while the variance estimated by traditional model lacks consistency in these conditions. Additionally, the variance estimated by the Lie group error model under static conditions is smaller than the traditional model. We can conclude that the Lie group model has more stability than the traditional model in the variance estimation of integrated navigation.

## 5. Experiment and Analysis in the Condition of Large Misalignment Angle

Accurate initial navigation parameters (especially the initial attitude angle) are the basis for SINS, and even integrated navigation, to accomplish tasks.

To research the necessity of Lie group error model based on Euler angles in large initial misalignment angle, in this section, SINS/GNSS and SINS/DVL are subjects, and the performances of the SO model, LSE model, and RSE model under different accuracy levels are quantitatively analyzed.

### 5.1. SINS/GNSS

**Figure 4:**
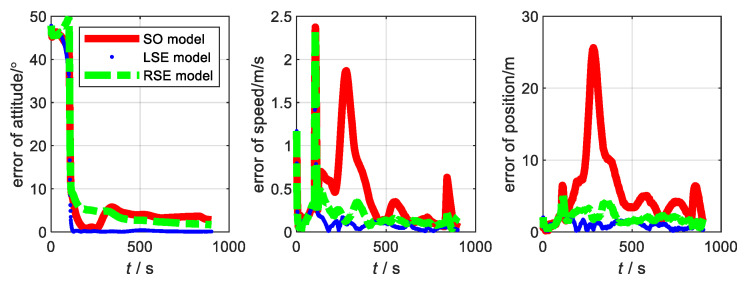
Error of each model at SINS/GNSS of low accuracy level.

**Figure 5:**
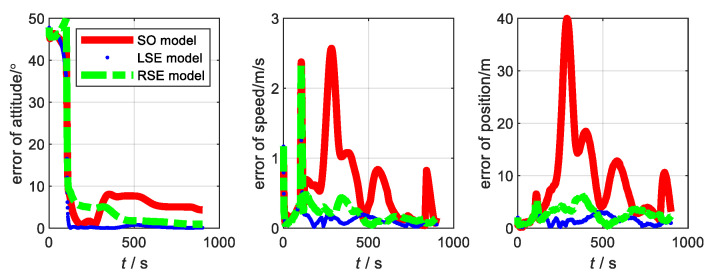
Error of each model at SINS/GNSS of medium accuracy level.

**Figure 6:**
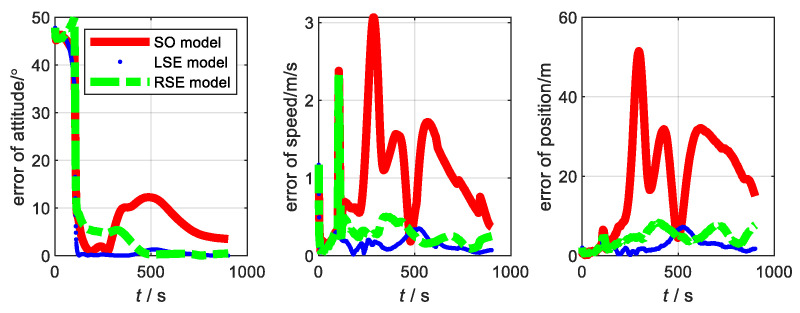
Error of each model at SINS/GNSS of high accuracy level.

The motion state of the carrier and the data fusion algorithm are the same as the flight experiment in Section 3. Considering that the aircraft takes off from smooth ground, the yaw angle is the main factor affecting it. Thus, the initial misalignment angle is set to $\left[\begin{array}{ccc} 20^{\circ } & 20^{\circ } & 45^{\circ } \end{array}\right]$. The main parameters of SINS, with low, medium, and high precision, are shown in Table 2. The errors of the three models at three accuracy levels are shown in Figure 4, Figure 5 and Figure 6, respectively. The root-mean-square errors (RMSE) [38] of the three models at different accuracy levels are shown in Table 3, Table 4, and Table 5, respectively. The errors of the three models at the three accuracy levels can be calculated by the following equations.

(34) $$\varphi_{error}=\sqrt{{\left(\theta_{error}\right)}^{2}+{\left(\gamma_{error}\right)}^{2}+{\left(\psi_{error}\right)}^{2}}$$

(35) $$v_{error}^{n}=\sqrt{{\left(v_{E,}^{n}{}_{error}\right)}^{2}+{\left(v_{N,}^{n}{}_{error}\right)}^{2}+{\left(v_{U,}^{n}{}_{error}\right)}^{2}}$$

(36) $$p_{error}^{n}=\sqrt{{\left(L_{error}\right)}^{2}+{\left(\lambda_{error}\right)}^{2}+{\left(h_{error}\right)}^{2}}$$

where $L_{error}$ and $\lambda_{error}$ are converted to meters.

From the flight experiment, we can see that the navigation accuracy and convergence of the LSE model and RSE model are better than that of SO model in the condition of large misalignment angle. Additionally, the higher the accuracy level of the system, the more obvious the advantages of the Lie group error model. Among them, the LSE model has the best navigation performance. This is because SINS/GNSS is a left invariant observation and fits better with left error model [21,31]. Therefore, for SINS/GNSS, the Lie group error model has a higher application value than the traditional error model.

### 5.2. SINS/DVL

**Figure 7:**
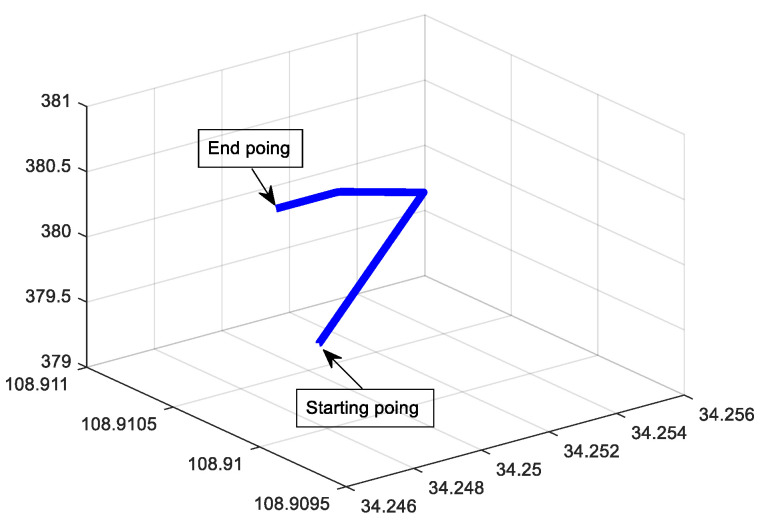
Trajectory of sailing.

**Figure 8:**
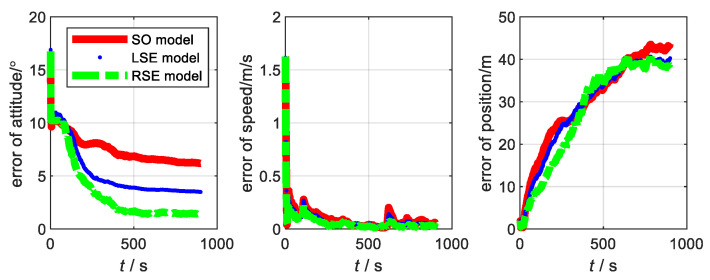
Error of each model at SINS/DVL of low accuracy level.

**Figure 9:**
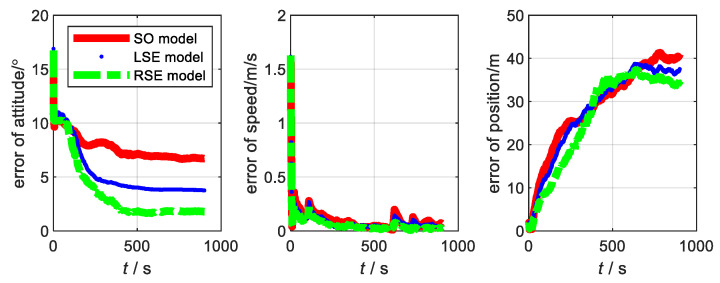
Error of each model at SINS/DVL of medium accuracy level.

**Figure 10:**
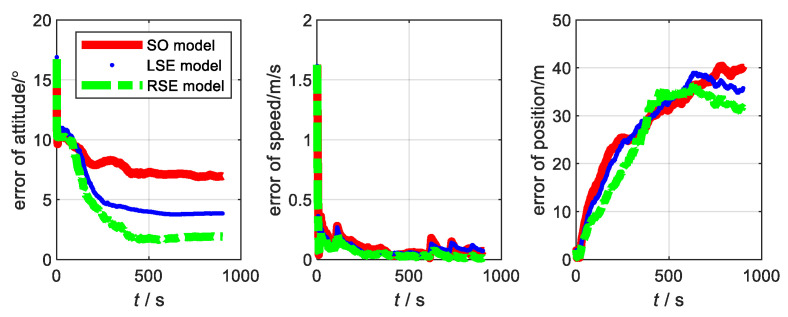
Error of each model at SINS/DVL of high accuracy level.

For SINS/DVL, we assume that the ship is sailing on the water surface, so that it only rotates around the *Z*-axis (Up), and the skyward speed is 0. When the ship starts sailing, the heave of the water surface is the main factor that affects it. The initial pitch and roll angles are used to represent the ups and downs; thus, the initial misalignment angle is set to $\left[\begin{array}{ccc} 20^{\circ } & 20^{\circ } & 10^{\circ } \end{array}\right]$. The main parameters of SINS with low, medium, and high accuracy and the data fusion algorithm are the same as (1). The trajectory of the sailing is shown in Figure 7. The corresponding errors are shown in Figure 8, Figure 9 and Figure 10, respectively. RMSE is shown in Table 6, Table 7 and Table 8, respectively.

From the results of the shipboard experiment, we can see that the accuracy of the LSE model and RSE model is better than that of the SO model. As the accuracy level increased, the results have some improvement. Among them, the RSE model is better than the LSE model, in terms of improvement. This is because SINS/DVL is closer to the right invariant observation [21]. Therefore, the Lie group error model is more applicable to SINS/DVL than the traditional model in the condition of misalignment angle.

Comparing the Lie group error model of integrated navigation based on the Euler angle and traditional error model [30], we can see that the traditional error model contains a large number of parameters related to the motion state of carrier and is more susceptible to the influence of the initial navigation parameters. However, due to fewer navigation parameters, the Lie group error model can reduce, or even avoid, the influence of the initial error.

In summary, the Lie group error model in conditions of large misalignment angles has certain research necessity.

## 6. Experiment and Analysis about Observability in Normal Condition

To analyze the observability of the SO model, LSE model, and RSE model, the field experiments of SINS/GNSS and SINS/DVL for the three models were conducted.

### 6.1. SINS/GNSS

**Figure 11:**
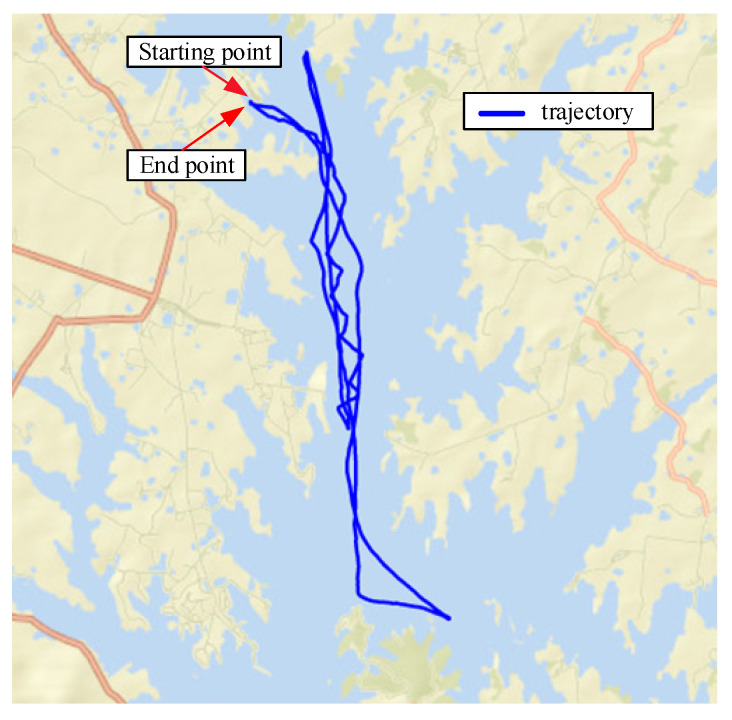
Experimental trajectory of SINS/GNSS.

**Figure 12:**
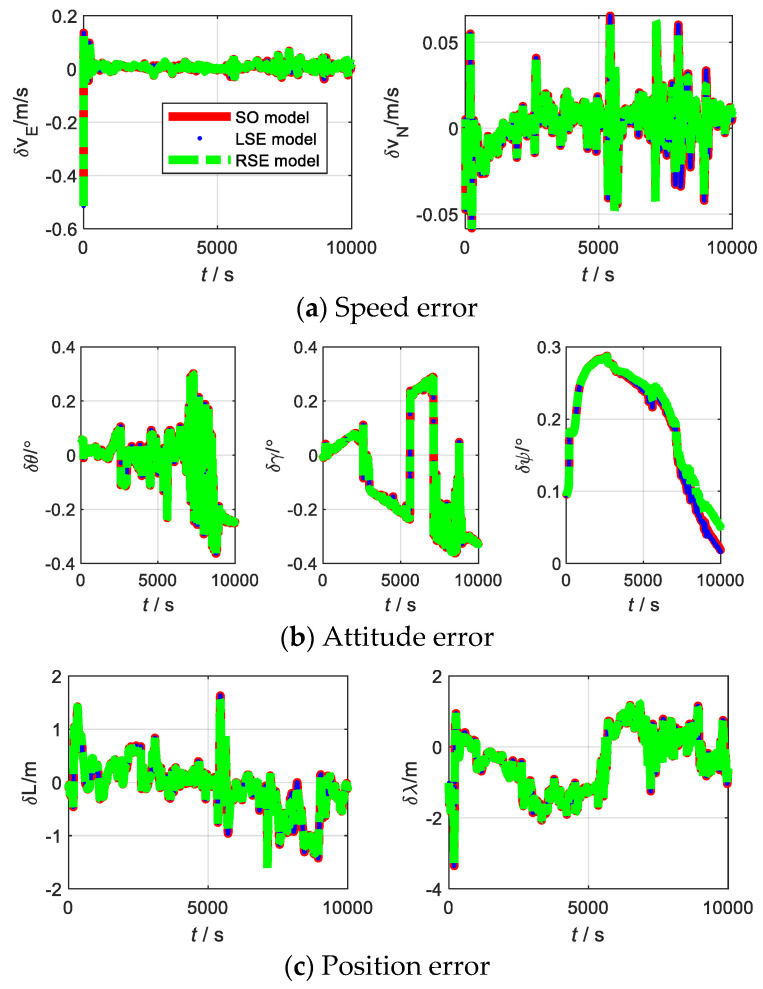
Errors of SINS/GNSS with different models.

The SINS/GNSS experiment was conducted at Mulan Lake, Wuhan, Hubei Province. The experimental equipment mainly includes: IMU, differential GPS, and a high precision integrated navigation module. IMU and differential GPS used the integrated navigation system, and the high precision integrated navigation module was used as a reference. The device parameters of IMU and differential GPS are shown in Table 9. The experimental trajectory is shown in Figure 11. The errors of the three models are shown in Figure 12. The corresponding RMSE is shown in Table 10. The observability of the three models at a moment is shown in Table 11.

From Figure 12 and Table 10, the estimation accuracy of the three models is not much different because the driving environment of the carrier is more stable. For SINS/GNSS, we can conclude that the Lie group model has the same applicability as the traditional model. From Table 11, the rank of the LSE model is higher than the RSE model and SO model. The amount of states that can be effectively estimated by the LSE model is more than the remaining two models. Comparing the singular values of the three models, we can find that the LSE model is generally larger and has a slightly better estimation effect. Although the estimation accuracy of the three models is almost the same, the observability of the LSE model is slightly better than the RSE model and SO model. Therefore, for SINS/GNSS, the LSE model has a higher practical performance.

### 6.2. SINS/DVL

**Figure 13:**
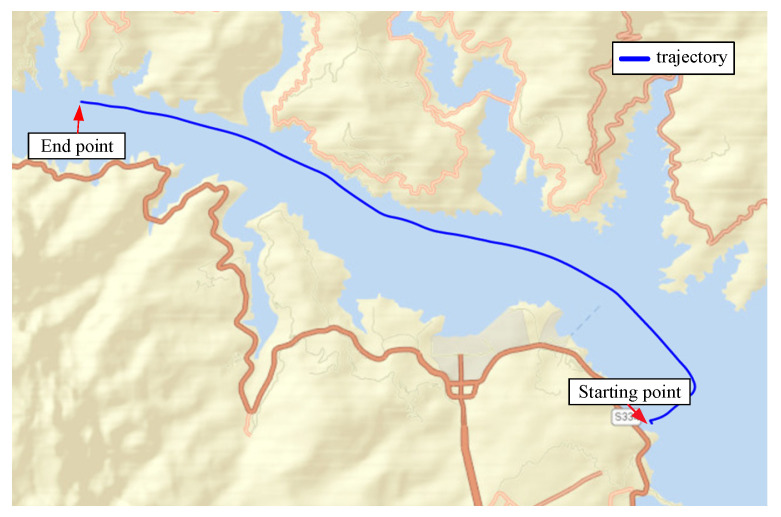
Experimental trajectory of SINS/DVL.

**Figure 14:**
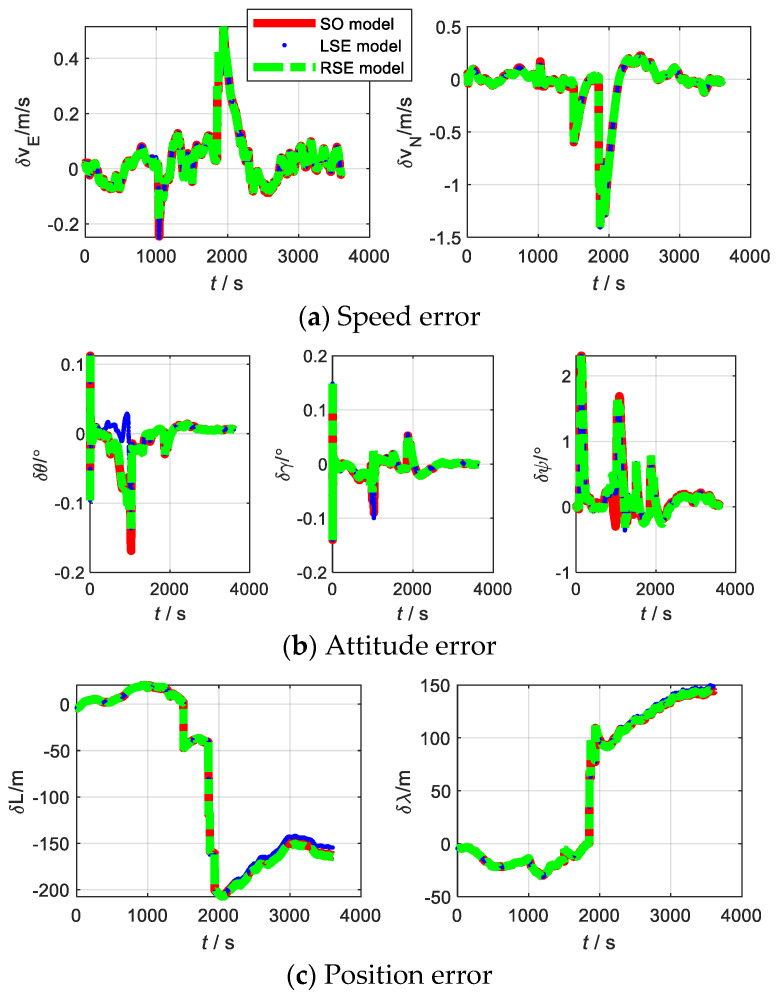
Errors of SINS/DVL with different models.

The experiment of SINS/DVL was conducted in Yangtze River, Badong County, Hubei Province. The experimental equipment mainly includes: IMU, DVL, and a high precision integrated navigation module. Among them, IMU and DVL were used for integrated navigation, and the high-precision integrated navigation module was used as a reference. The device parameters of IMU and DVL are shown in Table 12. The trajectory of the shipboard experiment is shown in Figure 13. The errors of the LSE model, RSE model, and SO model are shown in Figure 14, and the corresponding RMSE is shown in Table 13. The observability of SINS/DVL at a given moment is shown in Table 14.

From Figure 14 and Table 13, in the shipboard experiment, the estimation accuracy of the three models is almost the same. For SINS/DVL, the Lie group model has the same applicability as the traditional model. From Table 14, the LSE model, RSE model, and SO model have the same rank, indicating that the three models can effectively estimate the same number of states. Therefore, the number of states that can be effectively estimated is same for the three models. However, the singular values corresponding to each state of the RSE model are slightly larger than the LSE model and SO model. It shows that the estimation of the RSE model is slightly better than the remaining two models. Therefore, for SINS/DVL, the RSE model is more practical.

In summary, for integrated navigation, the observability and practicality of the Lie group model are better than the traditional error model.

## 7. Conclusions

In this paper, two commonly used integrated navigation models, SINS/GNSS and SINS/DVL, are used as subjects, the methods of analysis about necessity are introduced, and the PWCS matrix based on Lie group error model is established. From three perspectives, i.e., variance estimation, observability, and the estimation effect in the condition of misalignment angle, the specific advantages and the necessity of Lie group strapdown inertial integrated navigation error model based on Euler angle are demonstrated. The results of simulation and field experiments show that the Lie group error model has better variance estimation performance, navigation accuracy, and observability than the traditional error model. The work in this paper provides a basis for the research and application of the Li group model and broadens the ideas for the further research of integrated navigation models.

## Figures and Tables

**Table 1:** Relevant parameters of SINS.

Items	Drift	Frequency
Accelerometer	0.4 mg	10 Hz
Gyroscope	0.1°/h	

**Table 2:** Main parameters of the three accuracy classes of SINS.

Accuracy Level	Gyroscopic Drift	Accelerometer Drift
Low accuracy	0.1°/h	0.4 mg
Medium accuracy	0.01°/h	
High accuracy	0.001°/h	

**Table 3:** RMSE of the three models at SINS/GNSS of low accuracy level.

	Attitude (°)	Speed (m/s)	Position (m)
SO model	15.7519	0.6221	7.9800
LSE model	14.9156	0.1662	1.1990
RSE model	16.3242	0.2805	2.1713

**Table 4:** RMSE of the three models at SINS/GNSS of medium accuracy level.

	Attitude (°)	Speed (m/s)	Position (m)
SO model	16.2554	0.8437	12.7089
LSE model	14.9145	0.1814	1.7207
RSE model	16.2853	0.3039	3.0689

**Table 5:** RMSE of the three models at SINS/GNSS of high accuracy level.

	Attitude (°)	Speed (m/s)	Position (m)
SO model	16.9049	1.2108	23.1230
LSE model	14.9152	0.2125	3.0540
RSE model	16.2619	0.3534	5.1247

**Table 6:** RMSE of the three models at SINS/DVL of low accuracy level.

	Attitude (°)	Speed (m/s)	Position (m)
SO model	7.5717	0.1471	32.1127
LSE model	5.6684	0.1274	31.0728
RSE model	4.4067	0.1046	30.3764

**Table 7:** RMSE of the three models at SINS/DVL of medium accuracy level.

	Attitude (°)	Speed (m/s)	Position (m)
SO model	7.7692	0.1483	30.8575
LSE model	5.7301	0.1291	30.0482
RSE model	4.4725	0.1045	28.5841

**Table 8:** RMSE of the three models at SINS/DVL of high accuracy level.

	Attitude (°)	Speed (m/s)	Position (m)
SO model	7.8724	0.1495	30.1938
LSE model	5.7058	0.1351	29.7540
RSE model	4.4590	0.1047	27.7316

**Table 9:** Parameters of sensors in SINS/GNSS experiment.

Sensors	Items	Error Value	Frequency
IMU	Accelerometer	5 mg	200 Hz
	Gyroscope	0.003°/h	
Differential GPS	Position	0.1 m	1 Hz
	Speed	0.1 m/s	

**Table 10:** RMSE of SINS/GNSS with different models.

	Attitude (°)			Speed (m/s)		Position (m)	
	pitch	roll	yaw	eastward	northward	longitude	latitude
SO model	0.1222	0.2078	0.2116	0.0174	0.0141	0.4595	0.8638
LSE model	0.1222	0.2078	0.2113	0.0174	0.0141	0.4596	0.8638
RSE model	0.1217	0.2078	0.2165	0.0177	0.0138	0.4555	0.8631

**Table 11:** Observable analysis table for a moment in time of SINS/GNSS.

Model	SO Model	RSE Model	LSE Model
rank	11	11	12
Singular values	3.8753	3.8754	3.8753
	3.8729	3.8729	3.8729
	3.8729	3.8729	3.8729
	0.0836	0.0846	0.0836
	0.0008	0.0008	0.0008
	1.9160 × 10^−7^	3.1657 × 10^−8^	1.9399 × 10^−7^
	1.6197 × 10^−8^	2.7185 × 10^−8^	1.6177 × 10^−8^
	1.3934 × 10^−8^	3.1138 × 10^−9^	1.3934 × 10^−8^
	1.3667 × 10^−9^	6.1795 × 10^−10^	1.3520 × 10^−9^
	1.1747 × 10^−9^	2.0519 × 10^−10^	1.1743 × 10^−9^
	2.8258 × 10^−11^	7.8840 × 10^−12^	1.2359 × 10^−10^
			3.5975 × 10^−14^

**Table 12:** Sensors parameters of SINS/DVL experiment.

Sensors	Items	Error Value	Frequency
IMU	Accelerometer	0.05 mg	200 Hz
	Gyroscope	0.02°/h	
DVL	Position	10 m	1 Hz
	Speed	0.005 m/s	

**Table 13:** RMSE of SINS/DVL with different models.

	Attitude (°)			Speed (m/s)		Position (m)	
	Pitch	Roll	Yaw	Eastward	Northward	Longitude	Latitude
SO model	0.0273	0.0151	0.4386	0.1083	0.2475	86.2030	120.5530
LSE model	0.0159	0.0151	0.4098	0.1082	0.2497	88.1891	117.4460
RSE model	0.0274	0.0134	0.4314	0.1091	0.2489	86.4751	121.4989

**Table 14:** Observable analysis table for a moment in time of SINS/DVL.

Model	SO Model	RSE Model	LSE Model
Rank	8	8	8
Singular values	4.0971	4.1057	4.0779
	4.0970	4.1057	4.0663
	3.8753	3.8753	3.8755
	0.7313	0.7775	0.7363
	0.7308	0.7775	0.7347
	0.0836	0.0837	0.0836
	0.0073	0.0078	0.0073
	0.0073	0.0078	0.0073
	4.0971	4.1057	4.0779

## Data Availability

Not applicable.
